# Responses of germination, photosynthesis, and gene expression in four cotton varieties under drought stress

**DOI:** 10.3389/fpls.2025.1736650

**Published:** 2026-01-23

**Authors:** Jiazila Baha, Xiaohong Zhao, Yage Li, Xue Zhai, Xinchuan Cao, Weifeng Guo

**Affiliations:** 1 College of Agriculture, Tarim University, Alar, China; 2 Key Laboratory of Genetic Improvement and Efficient Production for Specialty Crops in Arid Southern Xinjiang of Xinjiang Corps, Tarim University, Alar, China

**Keywords:** cotton, drought resistance, gene expression, germination period, photosynthesis

## Abstract

**Background:**

Drought is a crucial abiotic stress factor affecting the growth and development of cotton, and there is significant differentiation in drought resistant among different cotton varieties. To reveal the drought resistance mechanisms of four cotton varieties, this study conducted a systematic analysis across multiple stages and multiple indicators.

**Methods:**

In this study, two drought resistant varieties (J206-5 and Jiumian 20) and two drought sensitive varieties (Xinluzhong 77 and Xinluzhong 67) were selected as experimental materials for seed germination, drought related gene expression during seedling stage, and photosynthetic indicators during flower and boll stage.

**Results:**

The six germination indicators of four cotton varieties showed a decreasing trend with the increase of PEG6000 concentration. Under 10% PEG6000 treatment, the relative germination potential, relative germination rate, relative germination index, and relative root fresh weight of drought resistant varieties were significantly higher than those of drought sensitive varieties. Under drought stress until July 19th, the Pn, Tr, and SPAD of drought resistant varieties were significantly higher than those of drought sensitive varieties. Under drought stress until August 19th, the Ci and SPAD of drought resistant varieties were significantly higher than those of drought sensitive varieties, and the Pn of drought resistant varieties was significantly higher than that of drought sensitive variety Xinluzhong 67. When drought resistant varieties were subjected to drought stress for 8d, except for the GhPPO-3 gene, the expression levels of the other 8 genes were significantly upregulated and reached their maximum values, and the expression levels were significantly higher than those of drought sensitive varieties.

**Conclusion:**

Under drought stress, drought resistant cotton varieties exhibit better seed germination, photosynthesis, and expression levels of drought related genes. This study provides a theoretical basis for cotton drought resistance breeding.

## 1 Introduction

Cotton is an important economic crop in Xinjiang, which is of great significance for increasing the income of local farmers and is also an important part of China’s economic development and food security. Drought is one of the main adverse factors affecting plant growth and crop yield (Perveen et al., 2022). In recent years, affected by the abnormal fluctuations of the global climate, the frequency of drought events has increased significantly, causing the problem of crop yield reduction, which has a huge impact on the economy of agricultural production (Saeed et al., 2024). In order to alleviate the adverse effects of drought and water shortage on cotton production, accelerating the cultivation of cotton varieties with high yield and drought resistance is the key and effective way to alleviate drought stress (Cattivelli et al., 2008). Therefore, it is of great significance to explore the drought resistance ability of cotton germplasm and cultivate drought resistant varieties to promote the sustainable and healthy development of cotton industry in Xinjiang (Baha et al., 2025).

Polyethylene glycol 6000 (PEG-6000) can reduce the water potential of the solution, limit the absorption of water by plant roots, and thus cause drought stress (Zhang et al., 2025). Research has shown that using different concentration gradients of PEG6000 to induce water stress environments yields the same effect as implementing varying degrees of drought stress in soil systems (Liu et al., 2020). Photosynthesis is also affected by water stress. The photosynthetic products of leaves are the material basis for crop growth (Yang et al., 2021). Drought alters photosynthesis, which affects the growth and development process of crops. Related studies have shown that drought stress can prolong seed germination time, slow down germination rate, reduce germination rate and related drought resistance index (Pei et al., 2014; Meng et al., 2022), and also lead to abnormal leaf morphology and decreased photosynthetic parameters in crops (Chen et al., 2023; Liu et al., 2023).

Drought stress induces the expression of a series of drought related genes in crops, among which the expression of most genes responds or tolerates drought stress at the cellular level (Bai et al., 2023; Du et al., 2024). The response of crops to drought stress is not the result of the independent action of a single gene, but rather the collaborative completion of a large number of genes through complex and diverse regulatory networks. The ultimate function of these networks is to help crops adapt to multiple environments. In the hierarchical gene regulatory network (GRN) model that regulates crop drought tolerance, upstream stress sensors transmit extracellular drought signals to intermediate transcription factors, which in turn regulate the expression of structural genes related to key drought resistance traits (Xu et al., 2023). Related studies have shown that drought stress can significantly induce physiological processes related to proline synthesis genes, peroxidase genes, catalase genes, ABA, and MDA to affect crop drought resistance (Tian et al., 2015; Xia et al., 2023).

On the basis of the findings of Baha et al. (2025) work, this study selected 2 drought resistant varieties (J206–5 and Jiumian 20) and 2 drought sensitive varieties (Xinluzhong 77 and Xinluzhong 67) for seed germination experiments using 10% and 15% PEG6000 solutions. Drought stress was applied during the seedling stage and the expression of drought related genes in leaves was detected. Drought treatment was carried out throughout the entire growth period in the field and photosynthesis of leaves was measured. By analyzing the germination indicators, photosynthesis, and expression levels of drought related genes of four varieties, theoretical references are provided to reveal the mechanism of cotton drought resistance.

## 2 Materials and methods

### 2.1 Materials

The experimental materials consisted of two drought resistant varieties (J206-5, Jiumian 20) and two drought sensitive varieties (Xinluzhong 77, Xinluzhong 67).

### 2.2 Methods

The seed germination experiment was conducted in an artificial climate chamber with a light intensity of 1400 umol/s/m^2^, day and night temperatures of 30°C and 21°C, day and night time of 16h and 8h, and relative humidity of 60%. The seeds were first disinfected with 7% sodium hypochlorite for 5 minutes, and then rinsed 3–5 times with distilled water. After disinfecting the culture dish with 75% alcohol, 2 layers of filter paper were placed in the dish, and 30 seeds were placed on the filter paper. Based on preliminary experiments, three treatments were set up, including distilled water, 10% and 15% PEG6000 solution, and each treatment was subjected to 3 biological replicates, 30 seeds per replicate. The height of the solution in the culture dish needs to exceed one-third of the seed height. A black cover was placed on the culture dish, and distilled water and PEG6000 solution were replenished daily.

The seedling experiment was conducted in an artificial climate chamber under the same conditions as the germination experiment. Two treatments were set up: drought stress and normal irrigation. Cotton seeds were sown in nutrient soil using seedling pots with small holes at the bottom (upper diameter of 12 cm, lower diameter of 10 cm, height of 10 cm), with a sowing depth of 2 cm. Twelve small seedling pots were placed in a large seedling pot with a transparent moisturizing cover, and 250 ml of Hogland nutrient solution was poured into each small pot. After flattening the cotyledons, the transparent cover was removed, and the seedlings were treated with continuous drought for 21 days, during which the nutrient solution was not irrigated, and the samples were collected after the treatment, the control seedlings were watered with 50 ml nutrient solution every 3 days. Leaves were collected on days 2, 4, 6, and 8 after the beginning of drought stress treatment, and at each time point, each treatment group, three leaves of each variety were collected for subsequent RNA extraction.

In 2025, the field experiment was conducted at the Tarim University experimental base, using a one film six row planting mode (10 cm+66 cm+10 cm+66 cm+10 cm), with each experimental plot being 2.27 m wide and 5 m long. Two treatments were set, normal irrigation and drought stress, the drought stress was imposed from June 10 to August 20. During this period, the control group was watered once a week (100%), and the drought treatment group was watered once every two weeks (50%).

#### 2.2.1 Determination of germination indicators

The germination standard is that the embryonic root breaks through the seed coat by 1 mm and the embryonic root is 1/2 of the seed length (Zhang et al., 2022). The measured seed germination indicators included germination potential, germination rate, germination index, embryonic root length, root fresh weight, root dry weight, and vitality index. The specific formulas were as follows:


Germination potential=number of germinated seeds in 3 days/number of tested seeds

Relative germination potential=treated germination potential/control germination potential

Germination rate=number of germinated seeds in 7 days/number of tested seeds

Relative germination rate=treated germination rate/control germination rate

$$\begin{array}{c} \text{Germination index}=\\ \sum (\text{Gt}/\text{Dt}),\text{ where Gt is the number of sprouts on day t and}\\ \text{ Dt is the corresponding number of germination days} \end{array}$$

Relative germination index=treated germination index/control germination index

Vitality index=germination index/root dry weight

Relative vitality index=treated vitality index/Control vitality index

Relative root fresh weight=treated root fresh weight/control root fresh weight

$$\begin{array}{c} \text{Relative embryonic root length}=\\ \text{treated embryonic root length}/\text{control embryonic root length} \end{array}$$

#### 2.2.2 Determination of photosynthetic indicators

Using the photosynthesis instrument Li-6400, the upper third leaf was measured on the mornings of July 19th and August 19th, 5 days after watering during the flowering period. Three individual plants were measured for each variety. The indicators included net photosynthetic rate (Pn), stomatal conductance (Gs), intercellular carbon dioxide concentration (Ci), and transpiration rate (Tr). Chlorophyll content (SPAD values) was measured using a chlorophyll analyzer (HM-YD), and the relative value of the data was calculated (drought stress/control).

#### 2.2.3 Total RNA extraction and cDNA synthesis

According to the protocol of the Column Plant Total RNA Extraction and Purification Kit (Bioengineering, China), total RNA was extracted from cotton leaves. The concentration and quality of RNA were detected using Nanodrop2000 (Thermo Fisher Scientific, USA). Samples (OD260/OD280 = 1.8~2.1) were subjected to experimental analysis. M-MuLV First Chain Kit (Bioengineering, China) was used to synthesize cDNA for real-time fluorescence quantitative PCR analysis.

#### 2.2.4 Real time fluorescence quantitative PCR analysis

Using a real-time fluorescence quantitative PCR instrument (Roche LightCycle)^®^ 96, Switzerland), based on the previously measured physiological experiments (Baha et al., 2025), the expression levels of genes involved in superoxide dismutase activity, proline synthesis, and malondialdehyde detoxification were analyzed. The sequences of superoxide dismutase related genes (GhMnSOD and GhSOD), proline synthesis related genes (GhGEL12, GhP5CS1, and GhP5CS2), and malondialdehyde detoxification related genes (GhcAPX, GhPPO, GhPPO-3, and GhPPO-9) were obtained from NCBI using cotton TUB7 as an internal reference gene( https://www.ncbi.nlm.nih.gov/). Real time fluorescence quantitative PCR was performed using a 2 × SG Fast qPCR Master Mix kit (Bioengineering, China) with a 24 μL reaction system, including 10 μL 2 × SG Fast qPCR Master Mix, 10 μL cDNA, 1 μL forward and reverse primers, and 2 μL ddH_2_O. Primer Premier 5 was used for RT qPCR primer design, and the primer information was shown in supplementary material (**Supplementary Table S1**). It was synthesized by Shanghai Shenggong Bioengineering Co., Ltd. Three leaf samples obtained from each variety, each treatment group and each time point need total RNA extraction and cDNA synthesis, respectively. Three cDNA templates were used as biological replicates, and the same cDNA template was added in parallel with 3 wells on the same qPCR plate for RT-qPCR reaction as technical replicates. The RT-qPCR reaction procedure was: pre denature at 95°C for 3 minutes; Denaturation at 95°C for 10 seconds; annealing at 50-60°C for 35 seconds, a total of 40 cycles. The relative expression levels of genes were calculated using the 2^-ΔΔCt^ method (Livak and Schmittgen, 2001), and the average values (drought stress/control) of drought resistant and drought sensitive varieties were plotted.

### 2.3 Data analysis

Microsoft Excel was used for data organization, and statistical analysis was performed using SPSS 20.0 for analysis of variance (ANOVA). The least significant difference (LSD) method at the P<0.05 level was applied to identify significant differences among treatments, and GraphPad Prism 10 software was used for charting.

## 3 Results

### 3.1 Seed germination characteristics of 4 cotton varieties

#### 3.1.1 Statistical analysis of germination traits under drought stress

**Figure 1:**
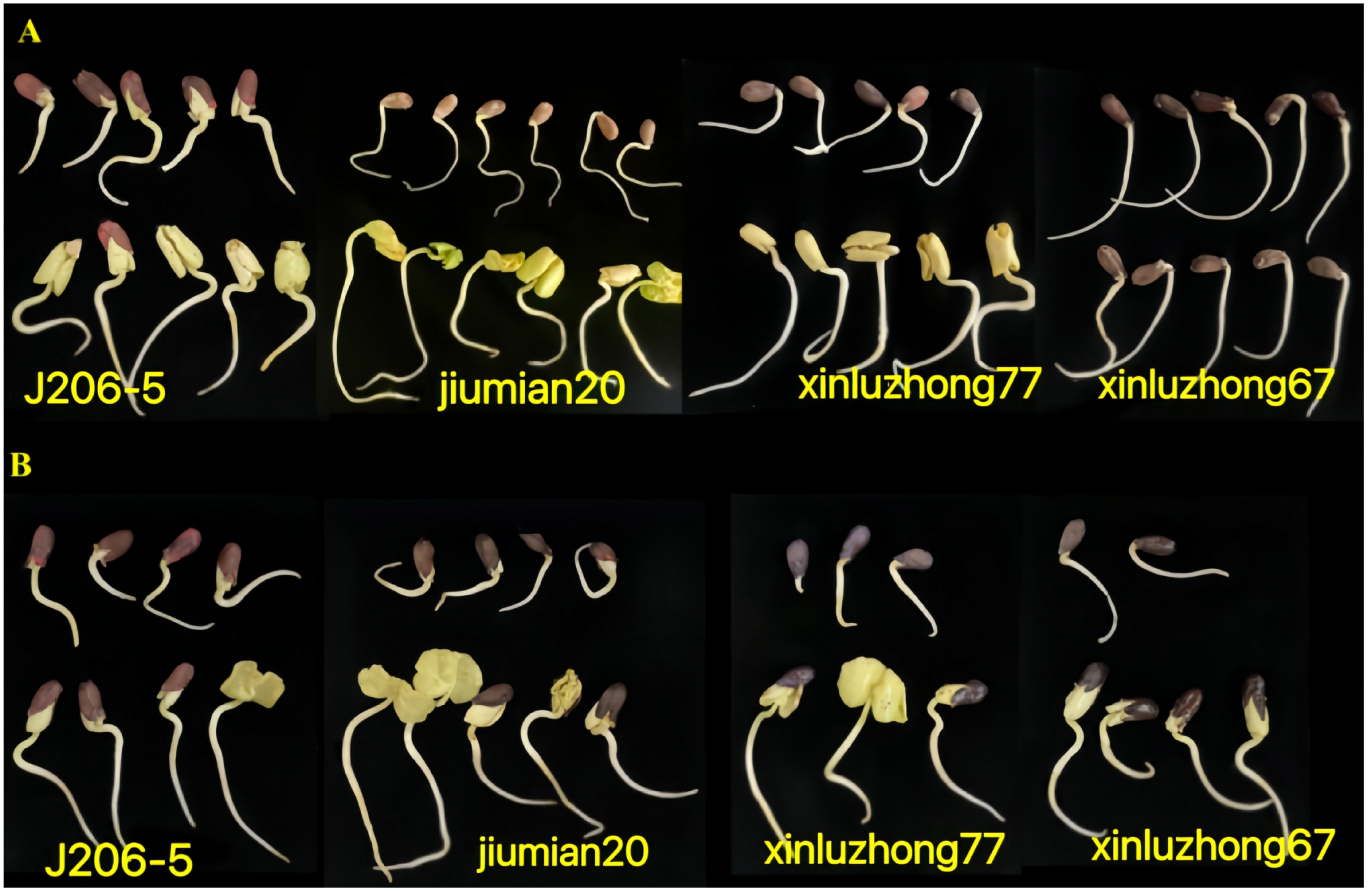
Germination of 4 cotton varieties treated with PEG6000 solution and distilled water for 96 hours. **(A, B)** represent the treatment with 10% and 15% PEG6000, respectively; The upper part of **(A, B)** shows drought stress, while the lower part shows distilled water treatment.

Four cotton varieties were shown to germinate in distilled water, 10% and 15% PEG6000 solutions for 96 hours (**Figure 1**). Compared to the control, the germination and growth of seeds treated with drought were inhibited, and drought sensitive varieties were more significantly inhibited.

The mean values of 6 germination indicators for 4 cotton varieties showed a decreasing trend with the increase of PEG6000 concentration (**Table 1**). There were significant differences in the relative germination index, relative root fresh weight, and relative vitality index between drought resistant varieties at 10% PEG6000. There were significant differences in the relative germination index, relative embryonic root length, and relative vitality index between drought sensitive varieties. There were no significant differences in the relative embryonic root length and relative vitality index between drought resistant and drought sensitive varieties, but the relative germination potential, relative germination rate, relative germination index, and relative root fresh weight of drought resistant varieties were significantly higher than those of drought sensitive varieties. At 15% PEG6000, five germination traits of drought resistant varieties were higher than those of drought sensitive varieties, and there were significant differences in three indicators.

**Table 1:** Statistical analysis of germination indicators of four cotton varieties treated with two PEG6000 solutions.

Varieties	PEG concentration	RGP	RGR	RGI	RRL	RRFW	RVI
J206-5	10%	0.82 ± 0.16a	0.84 ± 0.15a	1.47 ± 0.06a	0.93 ± 0.11a	0.62 ± 0.05a	1.60 ± 0.03a
Jiumian20		0.65 ± 0.07ab	0.73 ± 0.04ab	1.05 ± 0.14b	0.98 ± 0.07a	0.36 ± 0.01b	0.78 ± 0.11b
xinluzhong77		0.59 ± 0.04ab	0.59 ± 0.08ab	0.69 ± 0.19c	0.80 ± 0.02a	0.21 ± 0.02c	0.75 ± 0.04b
xinluzhong67		0.43 ± 0.01b	0.54 ± 0.13b	0.31 ± 0.18d	0.58 ± 0.08b	0.16 ± 0.03c	0.44 ± 0.09c
J206-5	15%	0.10 ± 0.03a	0.03 ± 0.01a	0.09 ± 0.01ab	0.58 ± 0.04a	0.15 ± 0.01a	0.18 ± 0.03ab
jiumian20		0.10 ± 0.02a	0.03 ± 0.02a	0.12 ± 0.04a	0.58 ± 0.02a	0.14 ± 0.04ab	0.22 ± 0.03a
xinluzhong77		0.06 ± 0.02a	0.03 ± 0.01a	0.09 ± 0.17ab	0.51 ± 0.02a	0.05 ± 0.01c	0.14 ± 0.04ab
xinluzhong67		0.09 ± 0.01a	0.03 ± 0.01a	0.06 ± 0.00b	0.54 ± 0.06a	0.08 ± 0.03bc	0.12 ± 0.03b

RGP, relative germination potential; RGR, relative germination rate; RGI, relative germination index; RRL, relative radicle length; RRFW, Relative root fresh weight; RVI, relative vigor index. (a–c) markers of significance of differences at 0.05 level.

#### 3.1.2 Effects of PEG6000 drought stress on six germination indicators

**Figure 2:**
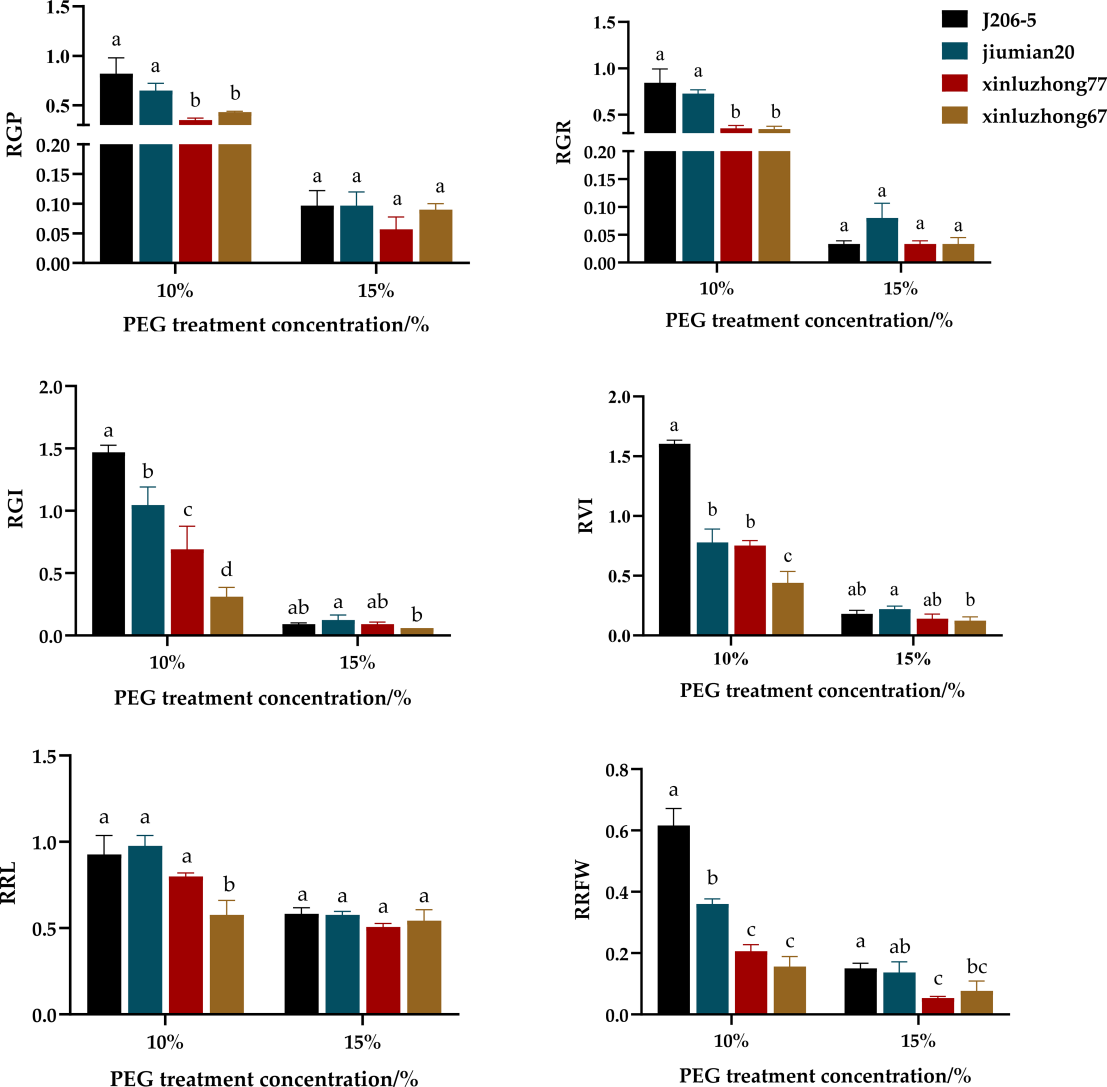
Effects of PEG6000 drought stress on 6 germination indicators of 4 cotton varieties. RGP, relative germination potential; RGR, relative germination rate; RGI, relative germination index; RVI, relative vigor index. RRL, relative radicle length; RRFW, Relative root fresh weight. (a–c) markers of significance of differences at 0.05 level.

At 10% PEG6000, the relative germination potential, relative germination rate, relative germination index, and relative root fresh weight of drought resistant varieties were significantly higher than those of drought sensitive varieties (**Figure 2**). The relative germination potential and relative germination rate of two drought resistant varieties were significantly higher than those of two drought sensitive varieties. Under 15% PEG treatment, there were no differences in relative germination potential and relative germination rate among four varieties (**Figure 2**).

Under 10% PEG treatment, the relative germination index of two drought resistant varieties was significantly higher than that of two drought sensitive varieties, and the relative vitality index of J206–5 was significantly higher than that of the two drought sensitive varieties. Under 15% PEG treatment, the relative germination index and relative vitality index of Jiumian 20 were significantly higher than those of drought sensitive variety Xinluzhong 67 (**Figure 2**).

Under 10% PEG treatment, relative embryonic root length of two drought resistant varieties was higher than that of Xinluzhong 67, and relative root fresh weight of two drought resistant varieties was significantly higher than that of two drought sensitive varieties. Under 15% PEG treatment, the relative root fresh weight of J206–5 was higher than that of two drought sensitive varieties (**Figure 2**).

### 3.2 Effects of drought stress throughout the entire growth period on photosynthesis in four cotton varieties

**Figure 3:**
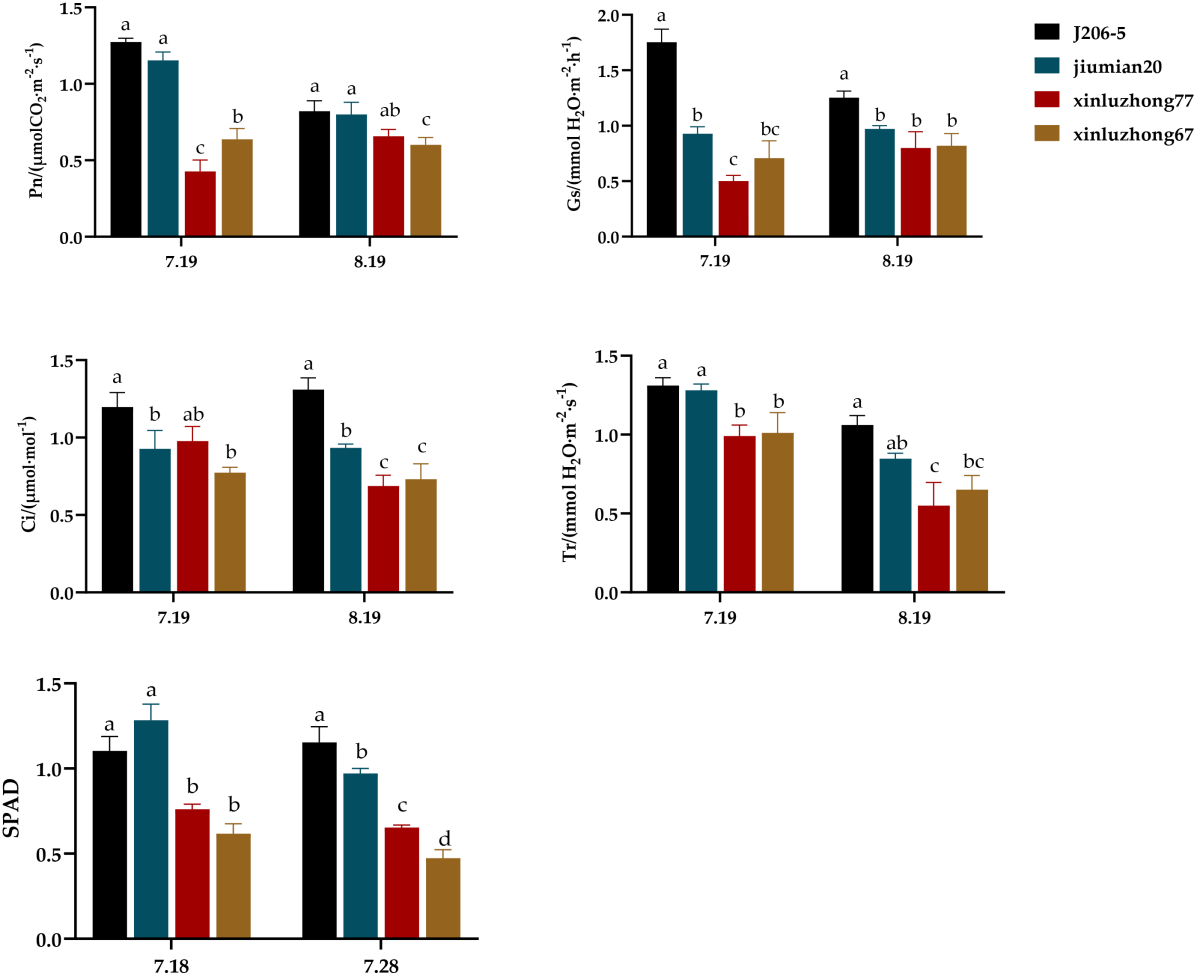
Effects of drought stress on photosynthesis in four cotton varieties. Pn, net photosynthetic rate; Gs, stomatal conductance; Ci, intercellular CO_2_ concentration; Tr, transpiration rate. 7.18, 8.19, 7.28 and 8.19 are the dates of photosynthesis measurement. (a–d) markers of significance of differences at 0.05 level.

The photosynthesis of four varieties was measured during the flower and boll period, and the relative values of net photosynthetic rate (Pn), stomatal conductance (Gs), intercellular CO_2_ concentration (Ci), transpiration rate (Tr), and SPAD were compared and analyzed (**Figure 3**). On July 19th, the Pn, Tr, and SPAD of two drought resistant varieties were significantly higher than those of two drought sensitive varieties. The Gs and Ci of J206–5 were significantly higher than those of drought sensitive variety Xinluzhong 67, and the Gs of Jiumian 20 was significantly higher than those of drought sensitive variety Xinluzhong 77. On August 19th, the Ci and SPAD of two drought resistant varieties were significantly higher than those of two drought sensitive varieties. The Pn of two drought resistant varieties was significantly higher than that of Xinluzhong 67, and the Gs and Tr of J206–5 were significantly higher than those of two drought sensitive varieties. The Tr of Jiumian 20 was significantly higher than that of drought sensitive variety Xinluzhong 77. In summary, under drought stress, drought resistant varieties exhibited better photosynthesis compared to drought sensitive varieties.

### 3.3 Gene expression analysis of four cotton varieties under drought treatment during seedling stage

#### 3.3.1 Expression analysis of superoxide dismutase synthesis related genes under drought stress

**Figure 4:**
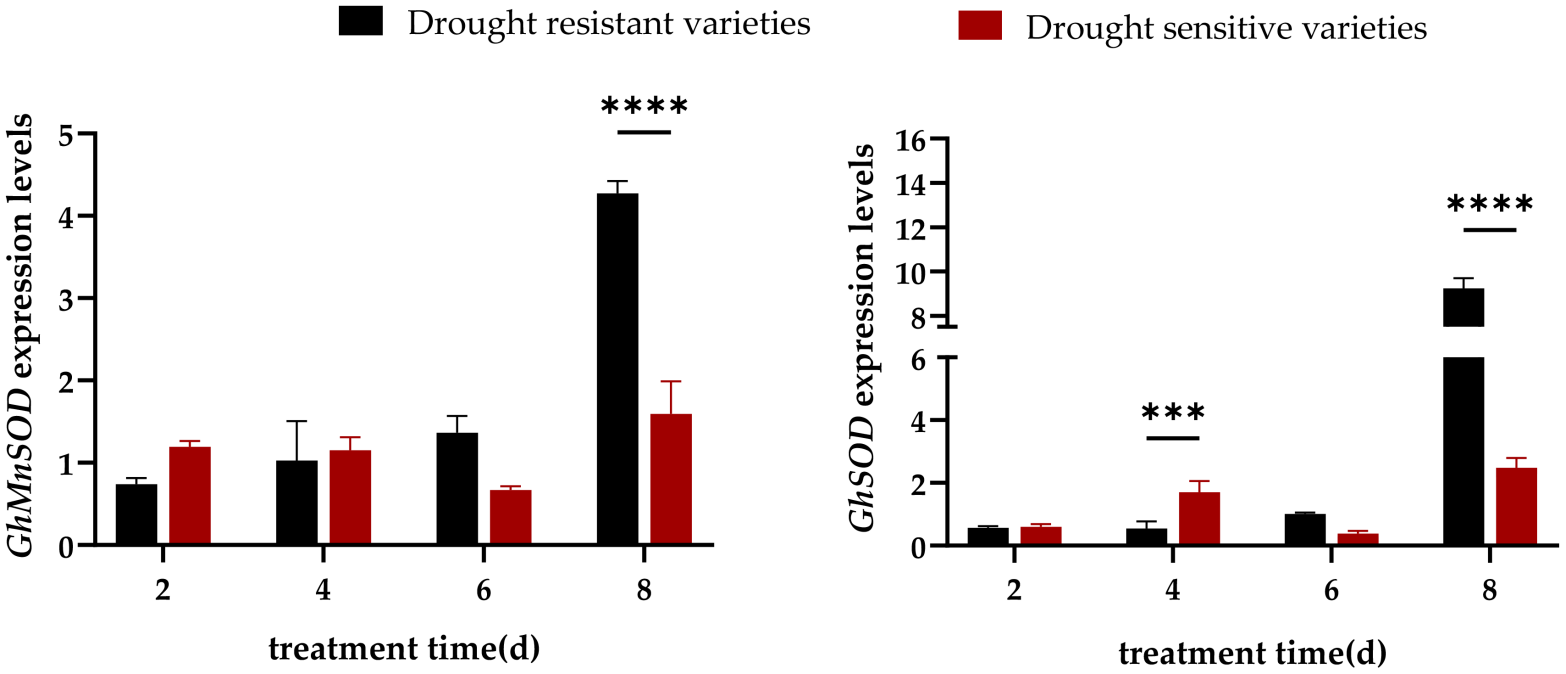
Expression analysis of genes related to superoxide dismutase synthesis in drought resistant and drought sensitive varieties under drought stress. Note: *** and **** are significant levels of 0.001 and 0.0001, respectively.

The expression levels of GhMnSOD and GhSOD in drought resistant and drought sensitive varieties showed an upregulation trend with the prolongation of drought treatment time, and the upregulation of GhMnSOD and GhSOD expression levels in drought resistant varieties was more significant (**Figure 4**). The expression levels of GhMnSOD and GhSOD in drought resistant varieties were significantly higher than those of drought sensitive varieties at day 8, with expression levels upregulated to 2.69-fold and 3.70-fold of the drought sensitive varieties, respectively. The expression levels of GhSOD in drought sensitive varieties were significantly higher than those of drought resistant varieties at day 4, with expression level upregulated to 3.15-fold of the drought resistant varieties. There was no significant difference in expression levels between drought resistant and drought sensitive varieties at other time points. This indicated that GhMnSOD and GhSOD genes had a positive effect on cotton drought stress.

#### 3.3.2 Expression analysis of proline synthesis related genes under drought stress

**Figure 5:**
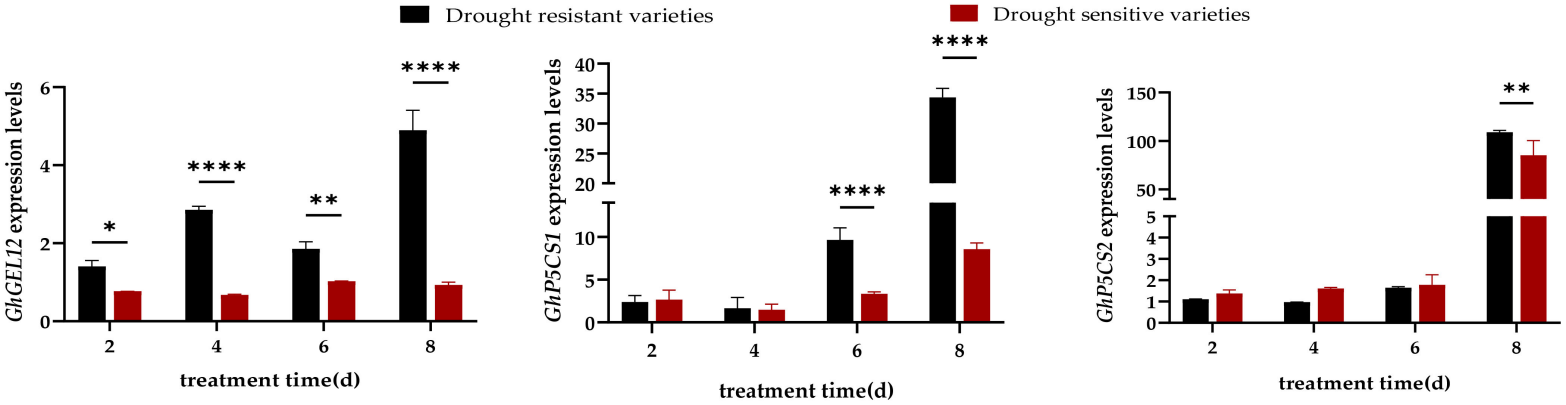
Expression analysis of proline synthesis-related genes in drought resistant and drought sensitive varieties under drought stress. Note: *, ** and **** are significant levels of 0.1, 0.01, and 0.0001, respectively.

The expression levels of GhGEL12, GhP5CS1, and GhP5CS2 in drought resistant varieties reached their maximum values at day 8, with expression levels were 4.89, 34.36, and 108.91, respectively. The expression levels of GhP5CS1 and GhP5CS2 in drought sensitive varieties showed an upward trend with the prolongation of drought treatment time, and the expression level of GhGEL12 did not change significantly (**Figure 5**).

The expression level of GhGEL12 in drought resistant varieties was significantly higher than that in drought sensitive varieties at all time points. The expression level of GhP5CS1 was significantly higher than that in drought sensitive varieties at 6d and 8d, with expression levels upregulated to 4.01-fold and 2.88-fold of the drought sensitive varieties, respectively, and the expression level of GhP5CS2 was significantly higher than that in drought sensitive varieties at 8d, with expression level upregulated to 1.28-fold of the drought sensitive varieties. This indicated that GhGEL12, GhP5CS1, and GhP5CS2 genes had a positive regulatory role in drought stress.

#### 3.3.3 Expression analysis of genes related to malondialdehyde detoxification under drought stress

**Figure 6:**
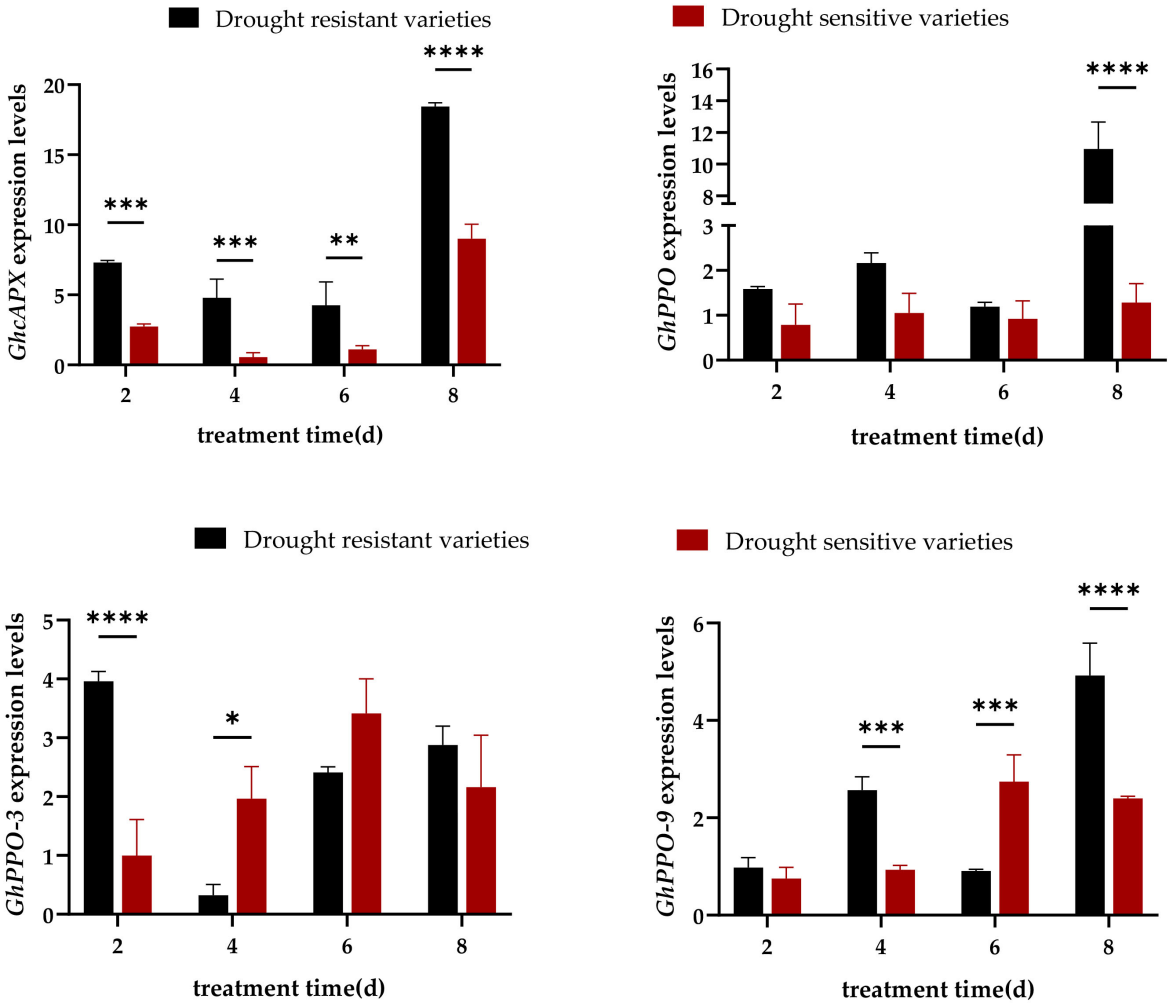
Expression levels of genes related to malondialdehyde detoxification production in drought resistant and drought sensitive varieties under drought stress. *, * *, * * *, * * * * are significant levels of 0.1, 0.01, 0.001, and 0.0001, respectively.

The expression levels of GhcAPX and GhPPO-3 in drought resistant varieties showed a trend of first decreasing and then increasing with drought treatment time. The expression levels of GhcAPX, GhPPO, and GhPPO-9 reached their maximum values at day 8, with expression levels were 18.43, 10.95, and 4.92, respectively. The expression level of GhcAPX gene in drought sensitive varieties showed a trend of first decreasing and then increasing. The expression levels of GhPPO-3 and GhPPO-9 reached their maximum values at day 6, with expression levels were 2.74 and 3.41, respectively. while the change in GhPPO expression level was not significant (**Figure 6**).

The expression level of GhcAPX in drought resistant varieties was significantly higher than that in drought sensitive varieties at all time points, the expression level of GhPPO was significantly higher than that in drought sensitive varieties at day 8, with expression level upregulated to 8.55-fold of the drought sensitive varieties, the expression level of GhPPO-3 was significantly higher than that in drought sensitive varieties at day 2, with expression level upregulated to 4-fold of the drought sensitive varieties, and the expression level of GhPPO-9 gene was significantly higher in drought resistant varieties from day 4 to day 8, with expression levels upregulated to 2.75-fold and 2.06-fold of the drought sensitive varieties, respectively, indicating that the expression levels of GhcAPX, GhPPO, GhPPO-3, and GhPPO-9 were higher in drought resistant varieties.

## 4 Discussion

### 4.1 Effects of drought stress on germination of drought resistant and drought sensitive cotton varieties

In the study of drought resistance of cotton during germination, PEG6000 solution was used to simulate drought stress, and seed germination studies have been conducted in oats, corn, peanuts, and other crops (Niu et al., 2021; Gao et al., 2024b; Cui et al., 2025). The results of this experiment showed that, at 10% PEG6000, the four germination indicators of drought resistant varieties were significantly higher than those of drought sensitive varieties. At 15% PEG6000, the relative germination index, relative vigor index and relative root fresh weight of drought resistant varieties were significantly higher than those of drought sensitive varieties (**Table 1**, **Figure 2**). This was similar to the findings of (Bai et al., 2023), who showed that compared to drought sensitive materials, drought resistant materials showed significantly increased relative germination potential, relative bud length, drought resistance index during germination, and vitality index of germinating seeds. In this experiment, the germination indicators of four cotton varieties decreased with increasing PEG concentration. At 15% PEG6000, various germination indicators were significantly inhibited (**Table 1**; **Figure 2**), which may be due to the decrease in soil water potential caused by drought, making it difficult for seeds to absorb water. Similarly, several studies have reported substantial changes in germination due to drought stress. For example, with the increase of PEG concentration, the relative germination potential, relative germination rate, relative germination index and other indicators of cotton seed were significantly reduced (Wang et al., 2009; Li, 2019; Dan et al., 2023), which was similar to the results of this study.

### 4.2 Effects of drought stress on photosynthesis during the flowering and boll stage of drought resistant and drought sensitive cotton varieties

The photosynthetic rate can reflect the strength of crop photosynthetic capacity and is one of the important indicators of the level of crop photosynthetic products. Chen et al. (2024) found that ZmKCS22 is localized to chloroplasts and cell membranes, and maintains the stability of thylakoid membrane structure by regulating chloroplast membrane lipid synthesis, avoiding the breakage of photosynthetic electron transport chains caused by drought. However, sensitive varieties have a higher degree of chloroplast membrane lipid peroxidation, and the photosynthetic mechanism is susceptible to damage. In this experiment, from drought stress until July 19th, values of Pn, Tr, and SPAD of drought resistant varieties were significantly higher than those of drought sensitive varieties. With the prolongation of drought stress time, the Tr and SPAD of four cotton varieties showed a decreasing trend, which was similar with the experimental result of (Gao et al., 2024a). From drought stress until August 19th, the Ci and SPAD of drought resistant varieties were significantly higher than those of drought sensitive varieties (**Figure 3**). This indicated that under drought conditions, the photosynthesis of crops decreased due to stomatal limitation, and the drought resistant varieties could ensure the accumulation of substances by maintaining higher photosynthetic efficiency. Drought resistant varieties regulate stomatal dynamic balance and reduce Tr to reduce water loss while ensuring carbon dioxide supply to adapt to the drought environment (Saeed et al., 2012; Perveen et al., 2019), and the stable accumulation of SPAD value can alleviate the damage of drought to photosynthetic system, which is not only the key index to measure the drought resistance potential of cotton, but also consistent with the physiological characteristics of drought resistant cotton to regulate antioxidant system activity, maintain chlorophyll synthesis and reduce photoinhibition. Therefore, the photosynthetic performance of the two drought resistant varieties was superior to that of the two drought sensitive varieties.

### 4.3 Effects of drought stress on gene expression related to drought resistant and drought sensitive cotton varieties during seedling stage

The response of crops to drought stress is not a single process, but a complex process composed of a series of physiological and molecular changes. Drought signals enter the nucleus through a complex signal transduction network, regulating transcription factor activity and specificity to alter gene expression and trigger adaptive changes in plants (Liu et al., 2025). For example, the promoter region of the cotton HVA22 gene is rich in ABRE (abscisic acid responsive elements). Under drought conditions, ABA signaling can rapidly activate GhHVA22E1D expression, thereby regulating the antioxidant enzyme system (Zhang et al., 2023). Luo et al. (2022) found that compared with normal conditions, drought stress considerably upregulated the expression levels of the antioxidant enzyme genes and proline synthesis genes. Li (2011) found that under drought stress, drought tolerant variety HI0466 induced the expression of SOD and POD enzyme genes, and showed strong drought resistance. These related reports are similar to the results of this experiment. In this experiment, drought stress significantly induced the expression levels of superoxide dismutase genes (GhMnSOD, GhSOD), proline synthesis genes (GhGEL12, GhP5CS1, GhP5CS2), and malondialdehyde detoxification genes (GhcAPX, GhPPO, GhPPO-3, GhPPO-9) in four resistant cotton varieties during the seedling stage. Research has shown that cysteine can induce the formation of drought resistant oat varieties, while increasing proline accumulation, AsA content, and the expression activity of CAT and POD antioxidant enzyme genes (Perveen et al., 2019). The experimental results showed that within 8 hours of drought stress, the expression levels of 8 genes in drought resistant varieties were significantly upregulated and significantly higher than those in drought sensitive varieties. Among them, the expression levels of GhGEL12, GhP5CS1, GhcAPX, and GhPPO in drought resistant varieties were higher than those in drought sensitive varieties at all four time points (**Figures 4**-**6**). Drought tolerant varieties enhanced their resistance to drought stress by upregulating the expression levels of antioxidant enzyme synthesis genes, proline synthesis genes, and malondialdehyde detoxification genes.

### 4.4 Response characteristics of cotton germination and photosynthetic indicators under drought stress

Drought stress significantly affects the germination and photosynthetic indicators of four cotton varieties (Shani et al., 2025b). Genotype × treatment interactions were also significant for most indicators, except Tr. The main effects of Ci and Gs treatment were not significant (**Supplementary Table S3**; **Supplementary Figure S7**), indicating that genetic differences in varieties and drought treatment are the core factors regulating these traits, and varieties have specific responses to drought. Principal component analysis (PCA) focuses on the first two main components PC1 and PC2 (Shani et al., 2025a), with a cumulative contribution rate of 85.335% (**Supplementary Table S4**). PC1 was mainly related to germination related indicators, while PC2 was mainly related to photosynthesis related indicators, indicating that crop germination and photosynthetic physiology under drought stress are relatively independent response modules. In summary, the germination and photosynthetic indicators of crops under drought stress can serve as the core dimensions for evaluating drought resistance, providing a basis for screening cotton stress resistant varieties.

## 5 Conclusion

The 6 germination indicators of 4 cotton varieties showed a decreasing trend with the increase of PEG6000 concentration. Under 10% PEG6000 treatment, the relative germination potential, relative germination rate, relative germination index, and relative root fresh weight of two drought resistant varieties were significantly higher than those of two drought sensitive varieties. The relative embryonic root length and relative vitality index of two drought resistant varieties were significantly higher than those of drought sensitive variety Xinluzhong 67. Under 15% PEG6000 treatment, the relative germination index and relative vitality index of drought resistant variety Jiumian 20 were significantly higher than those of drought sensitive variety Xinluzhong 67, and the relative root fresh weight of drought resistant variety J206–5 was significantly higher than that of drought sensitive variety Xinluzhong 77.

With the prolongation of drought stress time, the Tr and SPAD of four cotton varieties showed a decreasing trend. Under drought stress until July 19th, the Pn, Tr, and SPAD of drought resistant varieties were significantly higher than those of drought sensitive varieties. Under drought stress until August 19th, the Ci and SPAD of drought resistant varieties were significantly higher than those of drought sensitive varieties, and the Pn of drought resistant varieties was significantly higher than that of drought sensitive variety Xinluzhong 67.

Drought stress during the seedling stage upregulated the expression levels of superoxide dismutase genes (GhMnSOD, GhSOD), proline synthesis genes (GhGEL12, GhP5CS1, GhP5CS2), and malondialdehyde detoxification genes (GhcAPX, GhPPO, GhPPO-3, GhPPO-9) in four resistant cotton varieties. Within 8 days of drought stress, except for GhPPO-3 gene, the expression levels of other 8 genes in drought resistant varieties were significantly upregulated and reached their maximum values, and the expression levels were significantly higher than those in drought sensitive varieties.

The relative germination potential, relative germination rate, relative germination index, relative root fresh weight, Pn, Tr, SPAD and other indicators, as well as genes related to superoxide dismutase activity, proline synthesis, and malondialdehyde detoxification in this study provide reference for the screening of drought resistant germplasm.

## Data Availability

The original contributions presented in the study are included in the article/**Supplementary Material**. Further inquiries can be directed to the corresponding author.
