# Supplementary material for: Responses of germination, photosynthesis, and gene expression in four cotton varieties under drought stress
Source: Front Plant Sci. 2026 Jan 23;16:1736650. doi: 10.3389/fpls.2025.1736650 (PMC12875954; doi:10.3389/fpls.2025.1736650)
Supplement: Supplementary file 1 [file Table1.docx]

Supplementary Material

**Table 1S.** Gene information and primer sequence.

| Gene name | Login number | Primer sequence (5 '—3')(Forward/Reverse) |
| --- | --- | --- |
| GhMnSOD-F | LOC107897935 | GAAGATGAATGCTGAGGGTG |
| GhMnSOD-R | LOC107897935 | ATGCCAAGCAAAGGAACTAA |
| GhSOD-F | LOC107897935 | ACGATGCTGCTGCCTCTGTC |
| GhSOD-R | LOC107897935 | CCTCCGCCGTTGAACTTGAT |
| GhGEL12-F | LOC107932771 | AAACGGGAAAGCCGATGAGA |
| GhGEL12-R | LOC107932771 | ATAAGCCGCACCACCGAACT |
| GhP5CS1-F | LOC107957998 | TGTCGGGACTCTGTTTCATC |
| GhP5CS1-R | LOC107957998 | ATCATCCTGAGCAGCGGTAA |
| GhP5CS2-F | LOC107940896 | ATTCGGTTCCCATCGGTTCT |
| GhP5CS2-R | LOC107940896 | ATCGTGCAAGCCATCGAGCT |
| GhcAPX-F | LOC107959060 | AAGTGTTACCCAACTGTGAGCG |
| GhcAPX-R | LOC107959060 | CGAGAAGTCTGACGGCGATAT |
| GhPPO-F | LOC107901676 | GTGGTGGTCAAGCAGTGGCG |
| GhPPO-R | LOC107901676 | CAGGTTCAGTCGGTGTCTCG |
| GhPPO-3-F | LOC107923401 | TGCTCACATCGCCGTTCACA |
| GhPPO-3-R | LOC107923401 | TCACGTCGCTTCTTCCCTTG |
| GhPPO-9-F | LOC107960202 | CTTTCGGTTTGCCGTTCTGG |
| GhPPO-9-R | LOC107960202 | TCGTTTCGTGTTCGTCCTCA |
| GhTUB7-F | 817504 | GAAGGCATTCCACCTGACCAAC |
| GhTUB7-R | 817504 | CTTGACCTTCTTCTTCTTGTGCTTG |


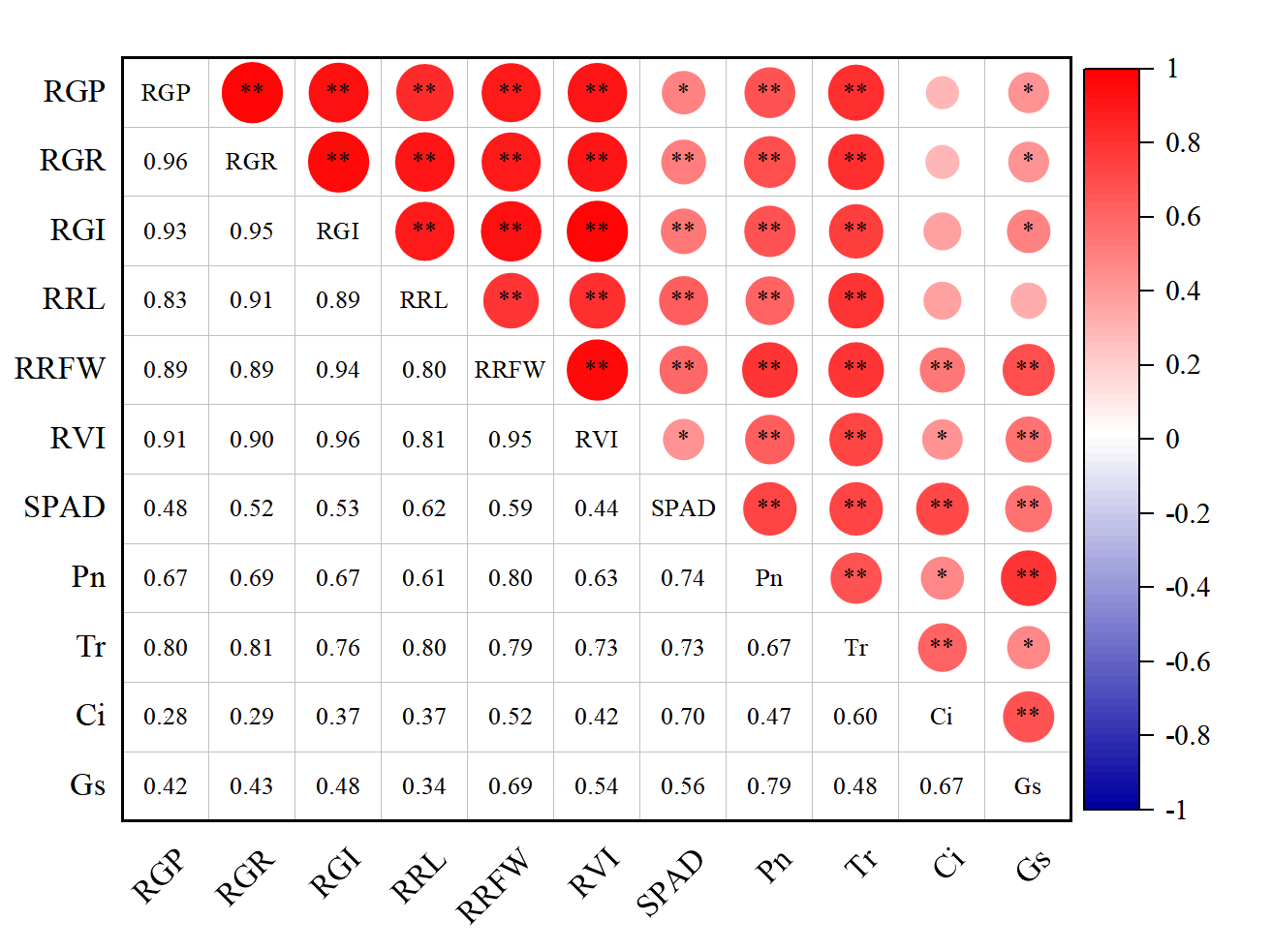


**Figure 7S.** Correlation analysis between eleven traits (Pearson correlation coefficient). Note: RGP, relative germinative potential; RGR, relative germination rate; RGI, relative germination index; RRL, relative radicle length; RRFW, Relative root fresh weight; RVI, relative vigor index; Pn, net photosynthetic rate; Tr, transpiration rate; Ci, intercellular CO2 concentration; Gs, stomatal conductance. ^*^ and ^**^ indicate significant correlations at the 0.05 and 0.01 level, respectively.

**Table 3S.** Variance analysis of eleven indicators under drought stress

| Source | DF | RGP | RGR | RGI | RRL | RRFW | RVI | Pn | Tr | Ci | Gs | SPAD |
| --- | --- | --- | --- | --- | --- | --- | --- | --- | --- | --- | --- | --- |
| G | 3 | 18.80^**^ | 31.56^**^ | 47.70^**^ | 16.32^**^ | 100.02^**^ | 112.85^**^ | 101.68^**^ | 30.20^**^ | 42.55^**^ | 82.85^**^ | 129.14^**^ |
| T | 1 | 328.66^**^ | 481.42^**^ | 453.58^**^ | 117.09^**^ | 345.63^**^ | 896.57^**^ | 37.40^**^ | 109.04^**^ | 2.50^ns^ | 0.07^ns^ | 24.10^**^ |
| G×T | 3 | 14.30^**^ | 27.16^**^ | 42.18^**^ | 10.75^**^ | 45.64^**^ | 97.75^**^ | 38.52^**^ | 1.54^ns^ | 6.40^**^ | 17.07^**^ | 8.13^**^ |
| Error | 16 |  |  |  |  |  |  |  |  |  |  |  |
| Total | 23 |  |  |  |  |  |  |  |  |  |  |  |

Note: RGP, relative germinative potential; RGR, relative germination rate; RGI, relative germination index; RRL, relative radicle length; RRFW, Relative root fresh weight; RVI, relative vigor index; Pn, net photosynthetic rate; Tr, transpiration rate; Ci, intercellular CO2 concentration; Gs, stomatal conductance. ^**^  indicate significant correlations at the 0.01 level, and ns (non-significant).

**Table 4S.** Principal component analysis of drought resistance coefficients for eleven indicators

| Indicators | Principal Component (PC) | |
| --- | --- | --- |
|  | PC1 | PC2 |
| Relative germination potential | 0.324 | -0.279 |
| Relative germination rate | 0.331 | -0.275 |
| Relative germination index | 0.334 | -0.231 |
| Relative radicle length | 0.314 | -0.209 |
| Relative root fresh weight | 0.344 | -0.035 |
| Relative vigor index | 0.326 | -0.199 |
| SPAD values | 0.258 | 0.385 |
| Net photosynthetic rate | 0.294 | 0.231 |
| Transpiration rate | 0.315 | 0.043 |
| Intercellular CO2 concentration | 0.206 | 0.547 |
| Stomatal conductance | 0.237 | 0.454 |
| Eigenvalue | 7.866 | 1.521 |
| Contribution % | 71.512 | 13.823 |
| Cumulative Contribution % | 71.512 | 85.335 |
